# Mr. Ring, on Diarrhœa and Cholera Morbus

**Published:** 1804-08-01

**Authors:** John Ring

**Affiliations:** New Street, Hanover Square


### *To the Editors of the Medical and Physical Journal.*

Gentlemen,

In pursuance of a plan I long ago commenced, I now transmit some miscellaneous remarks, which I beg the favour of you to insert. The first is relative to Diarrhœa and Cholera Morbus; diseases which, as Sydenham observes, are as sure to return at this season as swallows in the summer.

I have long been of opinion, that an erroneous theory, with regard to these complaints, has given rise to an erroneous practice. The bile is commonly supposed to be the principal cause of them; but different persons entertain different sentiments, respecting the particular manner in which the bile produces these disorders. Some ascribe it to a redundancy, others to a putridity, and others to an acescency of that fluid.

The last I believe to be only an imaginary alteration of bile, and that it never becomes acid. It frequently meets with more acid in the primæ viæ than it is capable of neutralising; and being evacuated with the other contents of the stomach or bowels in that state, it has given rise to this opinion.

Such an error would be of little consequence, did it not lead to a wrong practice. I have frequently known aperients continued in such cases long after the strength of the patient could bear them; and am perfectly convinced, by much experience, that in general they are not only unnecessary, but prejudicial in these complaints.

At the same time, it must be obvious to every one, that astringents should be proportioned to the disorder; and that whether they are exhibited before aperients, or after them in the usual manner, there is always a possibility of exceeding the bounds of moderation. But any practitioner who who is conversant in the treatment of such complaints must know, that when constipation follows a diarrhœa, it is easily removed, either by the oleum Ricini, magnesia vitriolata, or a clyster.

The following is a formula, which I have found equally pleasant and efficacious. ℞. Cretæ præp. ℈iv. Pulv. gum Arab. ℈ij. Aq. distill. ℥iv. Aq. cinnam. ℥iß. Sp. cinn. Syr. simpl. a. ʒij. M. To this I commonly add 30 drops of tinct. opii, and direct two large spoons full to be taken post s. l. s.

The following is a convenient form for a draught.

℞. Cretæ præp. ℈j. Pulv. gum Arab. gr. x. Aq. distill. ℥j. Aq. cinnam. ʒij. Sp. cinnam. Syr. simpl. a. ʒß. M. This is also a proper quantity to serve as a mixture for a child. The quantity of tincture for each purpose it is unnecessary to specify, since it must depend on the case.

I embrace this opportunity of pointing out an error in the London Pharmacopœia; or, at least, an alteration, which, I have been informed by an eminent physician, is an error.

When the Dispensatory was last revised, the gum Arabic, in the Mistura è Cretâ, was increased to eight times its former proportion. This, I am told, was a mistake; two ounces being directed, instead of two drachms. It was common, previous to this edition of the Dispensatory, to direct the composition to be made with double the former quantity of gum Arabic; but so remarkable a deviation as that alluded to, could not have arisen from design. It is fortunate the ingredient was a mild one; had it been otherwise, I should long ago have pointed out the supposed error.

I shall take the liberty of mentioning a method, by which the preparation of the Chalk Mixture is facilitated; a circumstance of no small moment, in some places, and at certain seasons, when autumnal complaints are prevalent. The method I follow is, to keep the prepared chalk and the gum Arabic in powder, mixed together in the proportion of two parts of the former with one of the latter, under the title of Pulvis Albus. Two drachms of this powder are sufficient for a six ounce mixture; and half a drachm for a draught, or for an ounce and half mixture for a child.

It must be obvious to every one conversant in practice, that a powder is a more convenient form of medicine for the poor, as well as for those who live at a distance, and cannot have a frequent supply; I shall therefore insert a compocomposition, which I have found extremely useful, and particularly during the late scarcity of provisions, when, owing to debility and improper foods, a diarrhœa was common, especially among the children of the poor.

℞. Cretæ præp. ʒxviij. Pulv. gum Arab. ʒix. Sacchari ℥ij. Pulv. cort. cinnam. ℈ij. Opii purif. ℈j. M.

I have sometimes substituted ginger for cinnamon, without any apparent disadvantage. This experiment was made for the sake of ascertaining, whether it might not be prepared in this manner, in any place where the expence of cinnamon, or of cassia, might be an object.

Those who have much practice among the poor must know, that a cheap medicine, which can be kept ready prepared, is an object of no small importance. The pulvis è cretæ comp. c. opio is of this kind; but it is not well adapted for children, on account of the bitterness of the tormentil.

Two drachms of this powder is a valuable present to a poor person, whose child labours under a diarrhœa. As much as will lie on a silver penny, or a seven shilling piece, may be given to an infant, in a little water; and repeated after every loose stool. A caution should be given not to add sugar.

This composition may be called Pulvis Astringens. I have known many hundred children apparently rescued from the grave by its salutary effect; and been amply repaid for the trifling expence of the composition by the gratitude of their parents. I therefore conceive it an indispensable duty to recommend the formula to others.

Were it generally adopted, or any better composition substituted, children would not so often be purged to death with rhubarb or magnesia; which, from the manner in which they are now used, or rather abused, may be considered as their bane.

To correct this very prevalent error, in some degree, I have recommended to many paupers, whose children laboured under slight diarrhœa, chalk instead of magnesia. This has been attended, in most instances, with a good effect; but it is sometimes insufficient, and the astringent powder is preferable.

I am far from being convinced, that rhubarb possesses the degree of astringency commonly ascribed to it. But if it be toasted, with an equal quantity of nutmeg, it becomes an excellent medicine as an astringent, and will sometimes succeed when all other astringents fail. The efficacy efficacy of this composition is increased by the addition of an opiate, or of any kind of spirit.

A person who had laboured under a diarrhœa for a long time, and been under the care of some of the most experienced medical practitioners, but in vain, was cured in a few days by a drachm of rhubarb and a drachm of nutmeg, toasted, and divided into three parts. Of these, one was taken every other morning, in brandy and water.

The most useful articles of food and drink that I have known in these complaints, when they occur in adults, are beef tea and cold brandy and water. Port wine is a popular, but a fallacious remedy; and in all probability, oftener does harm than good.

It is scarcely necessary to inform any medical man, that such persons as are troubled with this complaint, ought to abstain from vegetables, white meats, and malt liquor. But it is not unnecessary to inform the inexperienced practitioner, that unless he is very strict in his injunction, he will often be deceived in this respect, and be baffled in his attempt to cure his patient.

It must appear evident, from the manner in which I enlarge on this subject, that I think it of the greatest importance. A diarrhœa is one of the most frequent complaints, and attended with danger when it occurs in children, or in others of a weak constitution; and it is well known, that such are most liable to the disorder.

Various reasons are sometimes assigned for exhibiting aperients in this complaint, and even for repeating them, when there is no just reason for the practice; and the remedy is frequently worse than the disease. Among other reasons assigned, are those respecting the colour or the fetor of evacuations. These, however, in a real diarrhœa, are only imaginary reasons for administering a cathartic.

A fetor of the stools is frequently ascribed to a redundancy of bile; but surely this is an error, since it is acknowledged by the best judges, that the bile is of an antiputrescent nature; and every practitioner must know, that when there is an obstruction of the biliary ducts, the stools are often remarkably fetid.

If, in a common diarrhœa, cathartics are not in general necessary, but rather hurtful, they are less necessary, and more hurtful, in a cholera morbus. It is a popular opinion, that such a complaint must not be suddenly stopped. It is not, in general, easy to stop it suddenly; I have, however, sometimes known it yield to a single dose of the astringent; and have observed, that the cure is full as permanent in such instances, as in those where the evacuation tion gradually ceases. This would not be the case, were the evacuation critical.

It must also be considered, that such disorders are most frequent when the habit is relaxed by a sudden accession of hot weather. Hence a fermentation takes place in the stomach; in consequence of which, and an increased irritability of the whole primæ viæ, the complaints in question are excited. This opinion is strengthened by the common observation, that fruit is the general cause of the disorder. Fruit, particularly that which abounds with an acid, is indeed a very common cause, but far from being the only one of this disease; hard and indigestible substances of any sort, produce a similar effect. But debility, induced by sudden or long prevailing heat, appears to be the principal predisposing cause.

One very common proximate cause of a diarrhœa is stale malt liquor, which has in some measure undergone the acetous fermentation. In this case, as well as in those occasioned by sour fruits, or by an acid generated in the primæ viæ, it is surely more rational to correct the acid, to check fermentation, and to warm and invigorate the system, than to exhaust the little remaining strength by a debilitating plan.

I have sometimes known emetics given in the cholera morbus, but with very ill success. Happily, they are too soon rejected to do much mischief; but they occasion delay. The more violent the vomiting or purging is, the more powerful means are necessary for subduing them; and the more necessary it is to employ such means without delay. In cases of extremity, many a patient would be lost, were not the remedy frequently repeated; but the necessity of such repetition depends on a variety of circumstances, particularly on the quantity retained by the stomach.

The most distressing symptom in a cholera morbus, both to the practitioner and the patient, is the vomiting. To remove this, the medicines before mentioned are not ill adapted; but there is nothing which I have known so readily succeed, in some cases, as a small quantity of brandy, given alone, or with a dose of tinct. opii, adapted to the nature of the case.

Large quantities of chicken-broth were formerly given to persons labouring under this complaint. Such a practice was probably the result of the humoural pathology; and founded on error. As far as I am able to judge, it is pernicious; and only protracts the disease. The patient should should rather abstain from drinking; to which he is in many cases prompted by violent thirst: but what he drinks, unless the quantity be very small, only aggravates the disorder.

This complaint is not so fatal as might be expected; but the sufferings of the patient are so great, as to demand every assistance that art can give. I many years ago communicated my sentiments on this subject to a physician, who has long superintended the publication of this work, with honour to himself, and advantage to the public. His ideas were different from mine; and similar to those commonly entertained by medical practitioners. It was, however, no small gratification to me, and no small confirmation of the propriety of the practice I had long pursued, that this gentleman, some time afterwards, acknowledged he had also tried it with advantage.

A remark made in the Medical Journal, on a practice somewhat analogous to this, in one part of America, proves that the learned Editor has had no reason to alter the favourable opinion he entertained of this mode of treating diarrhœa and cholera morbus. He has had the candour to acknowledge, in a very respectable society, that he first adopted the practice at my suggestion; and that he still found it prove successful. This I mention, because I am sensible what weight his opinion will have with the members of the medical profession.

It is not without great concern, I am compelled to be a daily witness of the injury resulting from a contrary practice; which I am confident, sends a multitude of victims to an untimely grave, particularly of infants. What adds to the mischief is, a very prevalent opinion that a diarrhœa is salutary; and particularly during the period of dentition. This is often carried so far, that children are supposed to be in no danger who cut their teeth with a purging. How absurd this opinion is, it is unnecessary to inform any one of experience and discernment. It is a vulgar error, of a most fatal kind. I have particularly remarked, that no children suffer more from dentition than those who labour under this complaint. It particularly attacks those who are already weak; rendering them still weaker; and it must be apparent to every person, that debility at the same time retards the growth of the tooth, and occasions irritability of the gums, as well as of the whole system. Hence the sufferings of an infant are augmented, and prolonged; and a fatal catastrophe is a frequent result.

A diarrhœa

A diarrhœa is often occasioned by given children cathartics after eruptive diseases; a practice which medical men in general acknowledge to be totally unnecessary; and I hope, in time they will discontinue what, they must perceive, is hurtful. It is also a common custom with the lower classes of the community, to give children aperients for heat of every species; whether it proceeds from teething, from a cold, from the season of the year, from close and crowded apartments, or from a feather-bed. This custom, indeed, is not confined to the poor: and it may be doubted whether, in modern times, magnesia has not killed more than the sword.

Purging medicines are also considered by the ignorant, (a numerous tribe) as indispensably necessary in every kind of cutaneous disorder; and nothing is more common in this metropolis, than to give a child magnesia, or a course of alteratives, for a bug-bite.

This is a species of eruption that deceives parents and practitioners more frequently than any other. It is, indeed, by far the most common eruption of any in great towns; and affords the ignorant and the malicious an excellent opportunity of abusing the cow-pox. They call it a humour; and pretend it is owing to vaccination. It assumes various forms: at first it is like the sting of a nettle, or a nettle rash; afterwards it resembles a miliary eruption; then, as it gradually declines, it resembles a common rash. It has, however, often been dignified with pompous names; and been the subject of many a grave consultation. It sometimes is pustulous, and mistaken for the small-pox; and on this, as well as many other accounts, is not to be considered as unworthy of notice in a Medical Publication. It principally affects those parts which are uncovered, as the face, neck, and the fore arm. The upper part of the arm is generally free from this eruption; which affords an easy method of detecting the nature of the complaint.

It is often mistaken for the itch; and sulphur is applied in vain, till winter comes and removes the cause of the distemper. I am, &c.

JOHN RING.

*New Street, Hanover Square, July 17, 1804.*

CASE

